# A local outbreak of iatrogenic botulism associated with cosmetic injections of botulinum neurotoxin-containing products, England, 2025

**DOI:** 10.2807/1560-7917.ES.2025.30.39.2500746

**Published:** 2025-10-02

**Authors:** Joseph Jasperse, Kate Wilson, Sana Akbar, Iain Hayden, Qudsia Naseem, Alison Worthington, Amii Coglan, Matt Hewson, Alan Young, Min Fang, Yvonne Liu, Joanne Darke, Vanessa Wong, Gauri Godbole, Gareth J Hughes

**Affiliations:** 1 Field Service North East and Yorkshire and Humber, UK Health Security Agency, Newcastle upon Tyne, United Kingdom; 2 UK Field Epidemiology Training Programme, UK Health Security Agency, London, United Kingdom; 3 North East Health Protection Team, UK Health Security Agency, Newcastle upon Tyne, United Kingdom; 4 County Durham and Darlington NHS Foundation Trust, Durham, United Kingdom; 5 Gastrointestinal Infections, Food Safety and One Health Division, UK Health Security Agency, London, United Kingdom; 6 Durham County Council, Durham, United Kingdom; 7 Criminal Enforcement Unit, Medicines and Healthcare products Regulatory Agency, London, United Kingdom; 8 Molecular Analysis, Analytical and Biological Sciences, Science and Research, Medicines and Healthcare products Regulatory Agency, South Mimms, United Kingdom; 9 Bacterial Vaccine Standards, Standards Lifecycle, Science and Research, Medicines and Healthcare products Regulatory Agency, South Mimms, United Kingdom

**Keywords:** Botulism, *Clostridium botulinum*, botulinum neurotoxin, iatrogenic, outbreak, Europe

## Abstract

In June 2025, 25 botulism cases were identified among recipients of botulinum neurotoxin-containing cosmetic injections in North East England. A case-control study indicated that cases were more likely to have attended two specific practitioners and received an unlicensed product (p < 0.001). Testing of seized product detected a potency (370 units/vial) that was higher than listed on its labelling (200 units/vial). Strengthened regulation of cosmetic procedures is necessary for mitigating public health risks, which are exacerbated by the availability of unlicensed products.

Iatrogenic botulism is caused by the systemic spread of an excessive or improperly administered dose of botulinum neurotoxin (BoNT) during medical or cosmetic procedures [1]. Symptoms manifest as descending symmetrical paralysis which can progress to respiratory distress and require mechanical ventilation. Outbreaks of iatrogenic botulism are rare but have been linked to counterfeit products as well as the use of licensed products for unapproved indications [2-4]. Here we report the investigation of an outbreak of iatrogenic botulism linked to cosmetic injections of BoNT-containing products in North East England.

## Outbreak detection

Botulism is a notifiable disease in England and clinicians must report suspected cases to the UK Health Security Agency (UKHSA). On 11 June 2025, the UKHSA was informed of nine patients presenting to two hospitals in North East England with symptoms consistent with botulism following recent cosmetic injections with BoNT-containing products. An Incident Management Team was established with representatives from the UKHSA, County Council, Medicines and Healthcare products Regulatory Agency (MHRA) and local hospitals. The UKHSA informed local clinicians of the patients on 12 June and issued a press release on 13 June advising recipients of BoNT-containing products to seek medical attention should specified symptoms develop [5].

## Case definition and characteristics

A probable case was defined as an individual who received a cosmetic injection with a BoNT-containing product from a practitioner in North East England since 1 May 2025, developed at least one compatible symptom within 4 weeks of injection and was diagnosed with botulism by a healthcare worker. Compatible symptoms included difficulty swallowing, difficulty breathing (including shortness of breath) and altered speech.

Twenty-five cases were identified with onset dates ranging from 18 May to 9 June 2025 (Figure). Of these, 21 received injections from one of two independent practitioners (hereafter referred to as Practitioners A and B) living in the same area of one county (County X). The median duration from injection to symptom onset was 4 days (interquartile range: 2–6 days).

**Figure:**
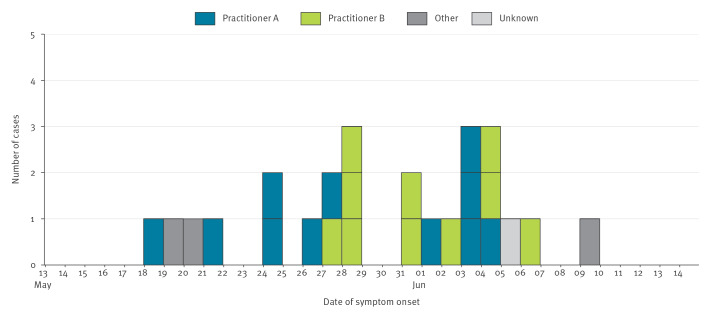
Epidemic curve of cases of iatrogenic botulism linked to cosmetic injections with botulinum neurotoxin-containing products by injecting practitioner, North East England, May–June 2025 (n = 25)

Most cases were females (22/25). Ages ranged from 25 to 82 years (median: 43 years). All cases attended a hospital emergency department and 16 received botulism antitoxin (Table 1). Serum samples from three cases were tested using a mouse bioassay and were negative for BoNT [6]. All cases survived.

**Table 1:** Selected characteristics of cases of iatrogenic botulism linked to cosmetic injections with botulinum neurotoxin-containing products, North East England, May–June 2025 (n = 25)

Characteristic	Number of cases	Percentage of cases
Age (years)		
Median	43	
Interquartile range	36–49	
Sex		
Female	22	88
Male	3	12
Symptoms^a^		
Blurred vision	23	92
Fatigue	23	92
Difficulty swallowing	22	88
Drooping eyelids	22	88
General weakness	21	84
Difficulty speaking	17	68
Change in sound of voice	15	60
Double vision	15	60
Dizziness	13	52
Dry mouth	12	48
Headache	12	48
Difficulty breathing	11	44
Numbness, tingling, chilling or burning sensation in skin anywhere on body	11	44
Coated or swollen tongue	10	40
Pain, swelling or bruising of injection site	7	28
Nausea	4	16
Abdominal pain	2	8
Constipation	1	4
Diarrhoea	1	4
Vomiting	0	0
Healthcare attendance^a^		
Attended hospital emergency department	25	100
Called NHS 111 (national medical advice line)	11	44
Attended general practitioner	10	40
Attended optician	3	12
Medical care received^a^		
Received botulism antitoxin	16	64
Admitted to hospital	16	64
Admitted to intensive care unit	1	4

NHS: National Health Service.

^a^ Cases could report more than one symptom and receive medical care from multiple locations.

All cases received cosmetic injections in the face and/or neck. Data on specific injection sites were available for 14 cases, for whom forehead, outer corners of the eyes (lateral canthal lines) and between the eyebrows (n = 13) were the most common sites. Injections were mainly administered in residential locations (practitioner’s home: n = 13; case’s home: n = 5; unspecified residence: n = 2) and five were given in a cosmetic clinic or salon.

## Case-control study

We conducted a case-control study with cases living in County X (21/25) to investigate risk factors for illness. Controls (n = 29) completed an online questionnaire advertised via County X Council’s social media. We defined a control as a County X resident aged ≥ 18 years who received a cosmetic injection with a BoNT-containing product since 1 May 2025 and developed either no symptoms or injection site pain only in the 2 weeks after injection.

Univariable odds ratios (ORs) and 95% confidence intervals (CIs) were calculated using Firth logistic regression [7]. For analysis of practitioners, cases and controls were restricted to individuals living within a 25-minute car journey from the centre point between Practitioners A and B, who lived less than three miles apart. We conducted a sensitivity analysis using journey times of 10, 15, 20 and 30 minutes.

Cases were significantly more likely to have attended Practitioners A or B (p < 0.001), reported receiving an unlicensed product (p < 0.001) and received more injections than at their previous appointment (p = 0.029) (Table 2). Receiving injections in ≥ 4 facial areas was also more common among cases, although was of borderline statistical significance (p = 0.058). The associations with Practitioners A and B remained significant across all journey times (all p < 0.01). No specific facial injection site was significantly associated with illness (data not shown).

**Table 2:** Univariable analysis from case-control study investigating potential risk factors for iatrogenic botulism linked to cosmetic injections with botulinum neurotoxin-containing products, North East England, May–June 2025

Exposure	Cases (n = 21)^a^		Controls (n = 29)^a^		Crude OR	95% CI	p value
	n	%	n	%			
Practitioner attended^b^							
Other	0	0	9	90	Reference		
Practitioner A	9	60	0	0	361.0	15.6–116,426	< 0.001
Practitioner B	6	40	1	10	82.3	5.5–13,282	< 0.001
Unknown	1		6		Not applicable		
Product received							
Licensed	5	36	11	100	Reference		
Unlicensed	9	64	0	0	39.7	3.8–5,480	< 0.001
Unknown	7		18		Not applicable		
Number of facial areas injected							
1–3 areas	3	23	16	55	Reference		
≥ 4 areas	10	77	13	45	3.7	1.0–17.0	0.058
Unknown	8		0		Not applicable		
Maximum number of injections in any one facial area							
1–3 injections	2	22	12	55	Reference		
≥ 4 injections	7	78	10	45	3.6	0.7–22.6	0.11
Unknown	12		7		Not applicable		
Number of injections vs previous appointment^c^							
About the same	5	50	16	84	Reference		
Fewer injections	0	0	1	5	1.0	0.0–21.9	1.0
More injections	5	50	2	11	6.6	1.2–46.7	0.029
Unknown	1		2		Not applicable		

CI: confidence interval; OR: odds ratio.

^a^ Numbers and percentages vary based on question completion rate. All questions were optional.

^b^ Analysis restricted to cases (n = 16) and controls (n = 16) living within 25 minutes car journey from the centre point between Practitioners A and B.

^c^ Analysis restricted to cases (n = 11) and controls (n = 21) who reported receiving a botulinum neurotoxin-containing product at least once before their most recent appointment.

We included questions about infection prevention and other good practices to provide insight into practitioner proficiency. Cases reported significantly lower practitioner compliance with all practices (p < 0.001; p = 0.02), except for use of disinfectant (Table 3).

**Table 3:** Participant-reported compliance of their cosmetic practitioner with selected good practices from case-control study of an outbreak of iatrogenic botulism, North East England, May–June 2025

Practice	Cases (n = 21)^a^			Controls (n = 29)^a^			p value^b^
	Responses	n	%	Responses	n	%	
Used disinfectant on injection site	13	10	77	29	27	93	0.16
Wore disposable gloves	13	8	62	29	27	93	0.02
Provided information about level of training	11	1	9	21	19	90	< 0.001
Provided consent form	13	0	0	29	25	86	< 0.001
Provided information about side effects	12	0	0	27	23	85	< 0.001
Washed hands before injection	13	2	15	29	22	76	< 0.001
Arranged pre-consultation with healthcare provider	14	0	0	29	16	55	< 0.001

^a^ Numbers and percentages vary based on question completion rate. All questions were optional.

^b^ Fisher's exact test.

## Environmental investigation

The MHRA inspected the wholesaler supplying Practitioner B and seized an unlicensed BoNT-containing product (hereafter referred to as Product X) which was received by 9 of 15 cases with product information available. Product X was tested using an endopeptidase immunoassay described previously [8]. Product samples were tested in duplicate on four replicate plates in a randomised layout alongside an in-house reference type A toxin and internal controls. The reference toxin was calibrated using the mouse lethality assay and had an assigned value of 1,831 mouse median lethal dose units/vial [9]. Sample potency estimates were calculated relative to the reference by EDQM CombiStats software (https://www.edqm.eu/en/lp-combistats) using parallel-line analysis.

Product X tested positive for BoNT type A with an estimated potency of 370 units/vial (95% CI: 327–419 units). The potency advertised on the product packaging was 200 units/vial. Subsequent investigation revealed that Product X had been imported from South Korea. Further supply chain investigations are underway.

## Discussion

Our investigation identified use of unlicensed BoNT-containing products and attendance at Practitioners A and B as potential risk factors for illness, although we cannot conclude definitively whether illness was attributable to the product(s), method of administration, or both.

Cases reported significantly lower practitioner compliance with several good practices. In England, BoNT-containing products must be prescribed by an authorised healthcare provider but there are no training or licensing requirements for individuals who administer them for cosmetic purposes. The UK government recently announced plans to introduce a licensing scheme that will require practitioners to comply with safety, training and insurance standards [10].

The estimated potency of Product X was 85% higher than listed on its packaging, although this value should be interpreted cautiously as potency testing methods vary by manufacturer and the values derived via different assays may not be comparable [11]. However, past studies have identified inaccurately labelled potency on other unlicensed products [12,13]. If potency was higher than advertised, there would be a risk of accidental overdose even when administered appropriately. Cases were more likely to report receiving more injections than their previous appointment, suggesting they may have also received a higher dose.

As Product X is unlicensed in the UK, there are no estimates available regarding its use. Several cases reported receiving Product X at a price substantially below that of licensed products. Internet searches indicate that Product X and other unlicensed BoNT-containing products are available for purchase from multiple online retailers without a prescription. The MHRA and UK Border Force have meanwhile announced the seizure of more than 4,700 vials of unlicensed BoNT-containing products across the UK since May 2023 [14]. The ongoing availability of cheaper, unlicensed products constitutes a considerable public health concern, particularly in the context of the expanding non-surgical cosmetic industry in the UK, where ca 900,000 BoNT injections are administered each year [15]. Although Product X was reported in only 9 of 15 cases with available information, this finding should be interpreted cautiously given the self-reported nature of the data and possibility of counterfeits [2,3].

Without laboratory confirmation, classification of iatrogenic botulism is difficult due to overlap between disease symptoms and adverse effects from BoNT-containing products. We encourage agencies investigating future cases to publish information on clinical presentation to build the evidence base for a probable case definition, which is not routinely used in Europe or the United States [16,17].

The UKHSA activated a national Incident Management Team to coordinate the response to further cases reported across England. Information about the incident has been shared via EpiPulse and other channels to raise awareness among local and international partners [18-20]. Thus far, we have not been informed of any cases from outside of the UK who have reported using Product X.

## Conclusion

Clinicians, public health and medicines regulatory agencies should be aware of the continued online availability of cosmetic BoNT-containing products with potencies that may not match package labelling. Individuals considering cosmetic procedures should be advised of the risks of using unlicensed products. Strengthened regulation of cosmetic procedures, such as that currently planned in the UK, is necessary for mitigating future public health risks.

## Data Availability

The data used in this investigation contain personal identifiable information. Pseudonymised information required to reproduce these results is available from the corresponding author on reasonable request.
